# Rituximab-containing reduced-intensity conditioning improves progression-free survival following allogeneic transplantation in B cell non-Hodgkin lymphoma

**DOI:** 10.1186/s13045-017-0487-y

**Published:** 2017-06-12

**Authors:** Narendranath Epperla, Kwang Woo Ahn, Sairah Ahmed, Madan Jagasia, Alyssa DiGilio, Steven M. Devine, Samantha Jaglowski, Vanessa Kennedy, Andrew R. Rezvani, Sonali M. Smith, Anna Sureda, Timothy S. Fenske, Mohamed A. Kharfan-Dabaja, Phillipe Armand, Mehdi Hamadani

**Affiliations:** 1 Division of Hematology and Oncology, Department of Medicine, Medical College of Wisconsin, Milwaukee, WI USA; 2 Center for International Blood and Marrow Transplant Research, Department of Medicine, Medical College of Wisconsin, Milwaukee, WI USA; 3 Department of Stem Cell Transplantation, MD Anderson Cancer Center, Houston, TX USA; 4 Division of Hematology-Oncology, Vanderbilt University Medical Center, Nashville, TN USA; 5 Division of Hematology, The Ohio State University Medical Center, Columbus, OH USA; 6 Division of Blood & Marrow Transplantation, Stanford University, Stanford, CA USA; 7 Section of Hematology/Oncology, The University of Chicago, Chicago, IL USA; 8 Servei d’Hematologica, Institut Català d’Oncologia, Hospital Duran i Reynals, Barcelona, Spain; 9 Department of Blood and Marrow Transplantation, H. Lee Moffitt Cancer Center and Research Institute, Tampa, FL USA; 10 Department of Medical Oncology/Hematologic Malignancies, Dana-Farber Cancer Institute, Boston, MA USA; 11 Center for International Blood and Marrow Transplant Research, Medical College of Wisconsin, 9200 W. Wisconsin Avenue, Suite C5500, Milwaukee, WI 53226 USA

**Keywords:** Rituximab, Reduced-intensity conditioning, Allogeneic hematopoietic cell transplant, Lymphoma

## Abstract

**Background:**

In B cell non-Hodgkin lymphoma (B-NHL), rituximab-containing reduced-intensity conditioning regimens (R-RIC) have been shown to provide favorable outcomes in single-arm studies; however, large multicenter studies comparing R-RIC and non-rituximab-containing reduced-intensity conditioning regimens (nonR-RIC) have not been performed. Using the CIBMTR database, we report the outcomes of R-RIC versus nonR-RIC regimens in B-NHL.

**Methods:**

We evaluated 1401 adult B-NHL patients undergoing allogeneic hematopoietic cell transplantation (allo-HCT) who received nonR-RIC (*n* = 1022) or R-RIC (*n* = 379) regimens. Graft-versus-host disease (GVHD) prophylaxis was limited to calcineurin inhibitor-based approaches.

**Results:**

Median follow-up of survivors in the R-RIC and nonR-RIC groups was 47 and 37 months, respectively. On multivariate analysis, no difference was seen between the R-RIC and nonR-RIC cohorts in terms of acute GVHD grade II–IV (RR = 1.14, 95%CI = 0.83–1.56, *p* = 0.43) or grade III–IV (RR = 1.16, 95%CI = 0.72–1.89, *p* = 0.54), chronic GVHD (RR = 1.15, 95%CI = 0.92–1.46, *p* = 0.22), non-relapse mortality (RR = 0.90; 95%CI = 0.67–1.22; *p* = 0.51), relapse/progression (RR = 0.79; 95%CI = 0.63–1.01; *p* = 0.055), and mortality (RR = 0.84, 95%CI = 0.69–1.02, *p* = 0.08) risk. However, R-RIC was associated with a significantly improved progression-free survival (RR = 0.76; 95%CI 0.62–0.92; *p* = 0.006). On subgroup analysis, mortality benefit was noted in the R-RIC group patients not receiving busulfan-based RIC (RR = 0.76; 95%CI = 0.60–0.96; *p* = 0.02) and with the use of a higher cumulative rituximab dose (RR = 0.43; 95%CI = 0.21–0.90; *p* = 0.02).

**Conclusion:**

Our analysis shows that inclusion of rituximab in RIC regimens improves progression-free survival in patients with B cell NHL. These data supports the use of R-RIC in B-NHL patients undergoing allo-HCT.

**Electronic supplementary material:**

The online version of this article (doi:10.1186/s13045-017-0487-y) contains supplementary material, which is available to authorized users.

## Background

Reduced-intensity conditioning (RIC) regimens currently account for approximately 40–45% of all allogeneic transplants performed in the USA [1]. These lower-intensity regimens rely more heavily on the graft-versus-tumor effects (exerted by the donor effector cells) to eradicate the residual disease in transplant recipients. RIC regimens are generally associated with a lower risk of procedure-related morbidity and non-relapse mortality (NRM) rates, thereby extending the option of allogeneic hematopoietic cell transplantation (allo-HCT) to patients with advanced age and/or significant medical comorbidities.

Considering the median age at diagnosis of patients with non-Hodgkin lymphomas (NHL) (~66 years), it is not surprising that RIC regimens now account for the majority of allo-HCTs performed for lymphomas in the USA [1]. However, the risk of disease relapse tends to be generally higher following RIC regimens compared to myeloablative allo-HCT in NHL [2, 3]. Identification of RIC approaches with the best risk/benefit ratio (NRM vs. relapse rate) in NHL remains an unmet medical need. Disease-specific RIC regimens, incorporating targeted therapies, can potentially provide improved peri-transplantation disease control, without increasing the rates of transplant-related morbidity and mortality. RIC regimens containing rituximab, an antiCD20 antibody with antineoplastic activity, have been employed in patients with B cell NHL [4–8]. Several single-institution studies incorporating rituximab in RIC regimens for B cell NHL have reported excellent disease control, with low rates of toxicity and severe graft-versus-host disease (GVHD) [4, 7]. However, large, multicenter studies comparing outcomes of RIC regimens incorporating rituximab, against those without rituximab in B cell NHL, have not been performed. We report here a registry analysis, comparing outcomes of rituximab-containing RIC (R-RIC) regimens versus non-rituximab containing RIC (nonR-RIC) regimens in B cell NHL.

## Methods

### Data sources

The CIBMTR is a working group of more than 500 transplantation centers worldwide that contribute detailed data on HCT to a statistical center at the Medical College of Wisconsin (MCW). Participating centers are required to report all transplantations consecutively, and compliance is monitored by on-site audits. Computerized checks for discrepancies, physicians’ review of submitted data, and on-site audits of participating centers ensure data quality. Observational studies conducted by the CIBMTR are performed in compliance with all applicable federal regulations pertaining to the protection of human research participants. The MCW and National Marrow Donor Program, Institutional Review Boards approved this study.

The CIBMTR collects data at two levels: Transplant Essential Data (TED) and Comprehensive Report Form (CRF) data. TED-data includes disease type, age, gender, pre-HCT disease stage and chemotherapy-responsiveness, date of diagnosis, graft type, conditioning regimen, post-transplant disease progression and survival, development of a new malignancy, and cause of death. All CIBMTR centers contribute TED-data. More detailed disease and pre- and post-transplant clinical information is collected on a subset of registered patients selected for CRF data by a weighted randomization scheme. TED- and CRF-level data are collected pre-transplant, 100-days and 6 months post-HCT, and annually thereafter or until death. Data for the current analysis were retrieved from CIBMTR (TED and CRF) report forms.

### Patients

Included in this analysis are adult (≥18 years) patients with B cell NHL, undergoing their first RIC or non-myeloablative conditioning (NMA) allo-HCT between 2008 and 2014. Eligible histologies included diffuse large B cell lymphoma (DLBCL), follicular lymphoma (FL), mantle cell lymphoma (MCL), and marginal zone lymphoma (MZL). Eligible donors including either HLA-identical sibling donors or unrelated donors (URD) matched at the allele-level at HLA-A, HLA-B, HLA-C and HLA-DRB1. Peripheral blood or bone marrow was permitted graft-source. GVHD prophylaxis was limited to calcineurin inhibitor (CNI)-based approaches. Patients receiving ex vivo graft manipulation (T cell depleted = 8 or CD34 selected grafts = 28) were not included. Use of antithymocyte globulin (ATG) during allo-HCT was not considered as an exclusion. Since alemtuzumab was used infrequently in patients receiving rituximab-based RIC (*n* = 1), these cases were not included. Radioimmunotherapy-based RIC regimens were excluded (*n* = 26).

### Definitions

The intensity of conditioning regimens was defined using consensus criteria [9]. Complete remission (CR) to last line of therapy before allo-HCT on CIBMTR forms is defined as complete resolution of all known areas of disease on radiographic assessments, while partial remission (PR) is defined as ≥50% reduction in the greatest diameter of all sites of known disease and no new sites of disease [10]. The resistant disease is defined as <50% reduction in the diameter of all disease sites, or development of new disease sites. Disease risk index (DRI) was defined as reported previously [11].

### Study endpoints

The primary endpoint was overall survival (OS); death from any cause was considered an event, and surviving patients were censored at last contact. NRM was defined as death without evidence of lymphoma progression/relapse; relapse was considered a competing risk. Progression/relapse was defined as progressive lymphoma after HCT or lymphoma recurrence after a CR; NRM was considered a competing risk. For progression-free survival (PFS), a patient was considered a treatment failure at the time of progression/relapse or death from any cause. Patients alive without evidence of disease relapse or progression were censored at last follow-up. Acute GVHD [12] and chronic GVHD [13] were graded using standard criteria. Neutrophil recovery was defined as the first of three successive days with absolute neutrophil count (ANC) ≥500/μL after post-transplantation nadir. Platelet recovery was defined as achieving platelet counts ≥20,000/μL for at least 3 days, unsupported by transfusion. For neutrophil and platelet recovery, death without the event was considered a competing risk.

### Statistical analysis

The R-RIC cohort was compared against the nonR-RIC cohort. Probabilities of PFS and OS were calculated as described previously [14]. Cumulative incidence of NRM, lymphoma progression/relapse, and hematopoietic recovery were calculated to accommodate for competing risks [15]. Associations among patient-, disease-, and transplantation-related variables and outcomes of interest were evaluated using Cox proportional hazards regression. A stepwise model building approach was used to identify covariates that influenced outcomes. Covariates with a *p* < 0.05 were considered statistically significant. The proportional hazards assumption for Cox regression was tested by adding a time-dependent covariate for each risk factor and each outcome. Interactions between the main effect and significant covariates were examined (in particular disease histology, GVHD prophylaxis regimen, and remission status at HCT), and none were found. Results are expressed as relative risks (RR). The center effect was examined using the random effect score test [16] for OS, PFS, relapse, and NRM. The marginal Cox proportional hazards model was used to account for the center effect on chronic GVHD, NRM, relapse, PFS, and OS [17]. Generalized linear mixed model with the logit link function was used to account for the center effect on acute GVHD II-IV and III-IV. The variables considered in multivariate analysis are shown in Additional file 1: Table S1. All statistical analyses were performed using SAS version 9.4 (SAS Institute Inc., Cary, NC).

## Results

### Baseline characteristics

The patient population was divided into two groups; 379 patients receiving rituximab as part of RIC regimens were included in the R-RIC group, while 1022 patients not receiving rituximab with conditioning constituted the nonR-RIC cohort. The baseline patient-, disease-, and transplantation-related characteristics are shown in Table 1. There was no significant difference between the two groups in terms of patient age, gender, race, Karnofsky performance score (KPS), HCT-comorbidity index (HCT-CI), interval between diagnosis and allo-HCT, median lines of prior therapy, remission status at HCT, total body irradiation (TBI) use in conditioning, donor type, donor-recipient sex match, and donor-recipient CMV serostatus. DLBCL was the most common histology in the nonR-RIC group (44%) while FL was the most common histology (37%) in the R-RIC group (*p* < 0.001). The proportion of patients with intermediate DRI (50 vs. 40%; *p* = 0.03) and history of a prior autologous HCT (43 vs. 26%; *p* < 0.001) was higher in the nonR-RIC cohort. Fludarabine/busulfan (Flu/Bu) ± total body irradiation (TBI) was the most common conditioning in the nonR-RIC group (35%) while fludarabine/cyclophosphamide (Flu/Cy) ± TBI in the R-RIC group (54%). CNI ± methotrexate-based GVHD prophylaxis was more common in the R-RIC group (55 vs 38%; *p* < 0.001). The graft source was mainly peripheral blood in both groups (95% [*n* = 970) vs. 98% [*n* = 371] in the nonR-RIC and R-RIC groups, respectively). Median follow-up of survivors was 37 months (range, 2–79 months) in the nonR-RIC group and 47 months (range, 6–89 months) in the R-RIC group.

**Table 1:** Baseline patient characteristics of patients with NHL reported to the CIBMTR from 2008 to 2014

Variable	Non-rituximab-RIC *N* = 1022	Rituximab-containing RIC *N* = 379	*p* value
Age at HCT, years			
Median (range)	57 (18–74)	56 (28–74)	0.32
>65	142 (14)	45 (12)	0.07
Male gender	667 (65)	248 (65)	0.95
Race			0.55
Caucasian	918 (90)	359 (95)	
African American	24 (2)	8 (2)	
Others	17 (2)	10 (3)	
Missing	63 (6)	2 (<1)	
Karnofsky performance score ≥90	622 (61)	235 (62)	0.24
HCT-CI			0.58
0	331 (32)	141 (37)	
1–2	301 (29)	115 (30)	
≥3	326 (32)	120 (32)	
Missing	64 (6)	3 (<1)	
Histology			<0.001
Follicular lymphoma	268 (26)	142 (37)	
Diffuse large B cell lymphoma	452 (44)	124 (33)	
Mantle cell lymphoma	274 (27)	106 (28)	
Marginal zone lymphoma	28 (3)	7 (2)	
Interval from diagnosis to HCT, months			0.73
Median (range)	36 (2-386)	39 (3-310)	
Median lines of therapy before HCT	3 (1-9)	3 (1-8)	0.54
Remission status at HCT			0.19
Complete remission	459 (45)	147 (39)	
Partial remission	421 (41)	169 (45)	
Chemorefractory	125 (12)	59 (16)	
Untreated	8 (<1)	2 (<1)	
Unknown	9 (<1)	2 (<1)	
Disease risk index at HCT			0.003
Low	405 (40)	184 (49)	
Intermediate	509 (50)	151 (40)	
High	99 (10)	42 (11)	
Missing	9 (<1)	2 (<1)	
History of prior autologous HCT	436 (43)	99 (26)	<0.001
Conditioning regimen			<0.001
Flu/Bu ± TBI	360 (35)	21 (5)	
Flu/Cy ± TBI	156 (15)	207 (54)	
Flu/Mel ± TBI	271 (27)	55 (15)	
2 Gy TBI ± Flu	196 (19)	40 (11)	
BEAM or similar	39 (4)	56 (15)	
TBI in conditioning	265 (26)	90 (24)	0.40
ATG in conditioning	191 (19)	75 (20)	0.64
Graft source			0.02
Bone marrow	52 (5)	8 (2)	
Peripheral blood	970 (95)	371 (98)	
Donor type			0.10
HLA-identical sibling	565 (55)	191 (50)	
URD 8/8	457 (45)	188 (50)	
Donor-recipient sex match	66 (36)	227 (46)	0.62
Male-male	434 (42)	168 (44)	
Male-female	222 (22)	89 (23)	
Female-male	233 (23)	80 (21)	
Female-female	133 (13)	42 (11)	
Donor/recipient CMV status			0.14
Both negative	305 (30)	112 (30)	
Either donor/recipient+	711 (70)	259 (68)	
Missing	6 (<1)	8 (2)	
Graft-versus-host disease prophylaxis			<0.001
Calcineurin inhibitor + MTX ± others^a^ (except MMF and sirolimus)	392 (38)	207 (55)	
Calcineurin inhibitor + MMF ± others^a^ (except sirolimus)	402 (39)	95 (25)	
Calcineurin inhibitor + sirolimus ± others^a^	200 (20)	75 (20)	
Calcineurin inhibitor alone	28 (3)	2 (<1)	
Median follow-up of survivors (range), months	37 (2–79)	47 (6–89)	

Number of centers that reported only nonR-RIC cases = 76, R-RIC cases = 3, and both (nonR-RIC and R-RIC) cases = 35

*Abbreviations*: *ATG* antithymocyte globulin, *Bu* busulfan, *CMV* cytomegalovirus, *Cy* cyclophosphamide, *Flu* fludarabine, *HCT* hematopoietic cell transplantation, *HCT*-*CI* HCT-comorbidity index, *Mel* melphalan, *MMF* mycophenolate mofetil, *MTX* methotrexate, *TBI* total body irradiation, *RIC* reduced-intensity conditioning

^a^For details, refer to Additional file 1: Table S8

### Hematopoietic recovery

The cumulative incidence of neutrophil recovery at day 28 was 97% (95%CI 96–98) in the nonR-RIC group compared to 98% (95%CI 96–99) in the R-RIC group (*p* = 0.51). The day-28 cumulative incidence of platelet recovery in similar order was 90 vs. 90% (*p* = 0.66; Table 2).

**Table 2:** Univariate analysis

	Non-rituximab-RIC *N* = 1022		Rituximab-containing RIC *N* = 379		
Outcomes	N eval	Prob (95%CI)	N eval	Prob (95%CI)	*p* value
ANC recovery >500/uL	1013		379		
28 days		97 (96–98)%		98 (96–99)%	0.51
100 days		99 (98–99)%		99 (97–100)%	0.99
Platelet recovery ≥ 20/uL	986		378		
28 days		90 (88–92)%		90 (87–93)%	0.66
100 days		96 (94–97)%		96 (93–97)%	0.79
Acute GVHD (II IV)	1004		377		
180 days		37 (34–40)%		43 (38–48)%	0.03
Acute GVHD (III IV)	1004		377		
180 days		13 (11–16)%		16 (12–19)%	0.29
Chronic GVHD	982		369		
1 year		44 (41–48)%		41 (36–47)%	0.32
2 years		53 (50–57)%		53 (47–58)%	0.79
NRM	988		367		
1 year		14 (12–16)%		13 (10–17)%	0.78
3 years		21 (18–24)%		20 (16–24)%	0.68
Relapse/progression	988		367		
1 year		26 (23–29)%		19 (15–23)%	0.004
3 years		32 (29–35)%		24 (20–29)%	0.005
PFS	988		367		
1 year		60 (57–63)%		68 (63–72)%	0.007
3 years		47 (44–50)%		56 (51–61)%	0.005
Mortality (inverse of OS)	1022		379		
1 year		70 (67–73)%		76 (71–80)%	0.04
3 years		56 (53–59)%		64 (59–68)%	0.01

Probabilities of neutrophil and platelet recovery, platelet recovery, acute GVHD, chronic GVHD, treatment-related mortality, and progression/relapse were calculated using the cumulative incidence estimate. Progression-free survival and overall survival was calculated using the Kaplan-Meier product limit estimate

*Abbreviations*: *ANC* absolute neutrophil count, *GVHD* graft-versus-host disease, *PROB* probability, *CI* confidence interval, *N eval* number evaluable, *NRM* non-relapse mortality, *PFS* progression free survival, *RIC* reduced-intensity conditioning, *OS* overall survival

### Acute and chronic GVHD

On univariate analysis, the cumulative incidence of grade II–IV acute GVHD at day 180 (Table 2) in the nonR-RIC cohort was 37% (95%CI 34–40), compared to 43% (95%CI 38–48) in the R-RIC group (*p* = 0.03). The corresponding rates of grades III–IV acute GVHD were 13% (95%CI 11–16) vs. 16% (95%CI 12–19) in the nonR-RIC and R-RIC groups, respectively, (*p* = 0.29). There was a significant center effect for grade II–IV acute GVHD (*p* = 0.004) and grade III–IV acute GVHD (*p* = 0.01). On multivariate analysis (Table 3), after adjusting for the center effect, R-RIC was not associated with a higher risk of grade II–IV (RR = 1.14, 95%CI = 0.83–1.56, *p* = 0.43) or grade III–IV (RR = 1.16, 95%CI = 0.72–1.89, *p* = 0.54) acute GVHD, relative to the nonR-RIC cohort. Grade II–IV acute GVHD was associated with a significantly high NRM (RR = 2.88, *p* < 0.0001), inferior PFS (RR = 1.6, *p* < 0.0001), and OS (RR = 1.93, *p* < 0.0001) in our study.

**Table 3:** Multivariate analysis results

	Number	Relative risk	95%CI lower limit	95%CI upper limit	*p* value
Acute GVHD (grades 2–4)^a^					
Non-rituximab RIC	1004	1			0.43
Rituximab-containing RIC	377	1.14	0.83	1.56	
Acute GVHD (grades 3–4)^a^					
Non-rituximab RIC	1004	1			0.54
Rituximab-containing RIC	377	1.16	0.72	1.89	
Chronic GVHD					
Non-rituximab RIC	982	1			0.22
Rituximab-containing RIC	369	1.15	0.92	1.46	
Non-relapse mortality					
Non-rituximab RIC	988	1			0.51
Rituximab-containing RIC	367	0.90	0.67	1.22	
Progression/relapse					
Non-rituximab RIC	988	1			0.055
Rituximab-containing RIC	367	0.79	0.63	1.01	
PFS					
Non-rituximab RIC	988	1			0.006
Rituximab-containing RIC	367	0.76	0.62	0.92	
Mortality					
Non-rituximab RIC	1022	1			0.08
Rituximab-containing RIC	379	0.84	0.69	1.02	

*Abbreviations*: *GVHD* graft-versus-host disease, *CI* confidence interval, *RIC* reduced-intensity conditioning

^a^Acute GVHD models used logistic regression

**Fig. 1:**
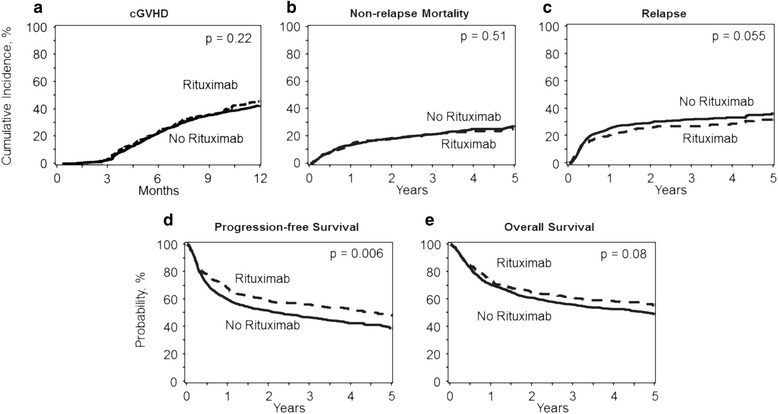
Adjusted transplantation outcomes of patients receiving R-RIC (interrupted lines) and nonR-RIC (solid line) regimens. **a** Cumulative incidence of chronic graft-versus-host-disease. **b** Cumulative incidence of non-relapse mortality. **c** Cumulative incidence of lymphoma relapse/progression. **d** Progression-free survival. **e** Overall survival

On univariate analysis, the cumulative incidence of chronic GVHD at 1 year (Table 2) after nonR-RIC transplant was 44% (95%CI 41–48) compared to 41% (95%CI 36–47) in the R-RIC group (*p* = 0.32). There was significant center effect noted for chronic GVHD (*p* < 0.0001). After adjusting for center effect, multivariate analysis (Table 3) showed no significant difference in the risk of chronic GVHD in the recipients of R-RIC transplant, (RR = 1.15, 95%CI = 0.92–1.46, *p* = 0.22) relative to the nonR-RIC group (Fig. 1a).

### NRM and relapse

The 1-year NRM rate in the nonR-RIC group was 14% (95%CI = 12–16) compared to 13% (95%CI = 10–17) in the R-RIC group (*p* = 0.78; Table 2). There was a significant center effect noted for NRM (*p* = 0.005). On multivariate analysis, after adjusting for the center effect, R-RIC was not associated with a higher risk of NRM (RR = 0.90, 95%CI = 0.67–1.22, *p* = 0.51) (Fig. 1b). Other factors independently associated with the risk of NRM are shown in the Additional file 1: Table S2.

The cumulative incidence of relapse/progression at 3 years was 32% (95%CI = 29–35) and 24% (95%CI = 20–29) in the nonR-RIC and R-RIC groups, respectively, (*p* = 0.005; Table 2). On multivariate analysis, the risk of relapse/progression was not significantly different between the two groups (RR = 0.79, 95%CI 0.63–1.01, *p* = 0.055) (Table 3) (Fig. 1c). Other factors independently associated with the risk of relapse/progression are shown in the Additional file 1: Table S2.

### Progression-free survival

The 3-year PFS was significantly better in the R-RIC group (56%; 95%CI = 51–61) compared to the nonR-RIC group (47%; 95%CI = 44–50; *p* = 0.005; Table 2). On multivariate analysis (Table 3), relative to the nonR-RIC group, the R-RIC was associated with a significantly improved PFS (RR = 0.76, 95%CI = 0.62–0.92, *p* = 0.006) (Fig. 1d). Other factors independently associated with the PFS are shown in the Additional file 1: Table S2.

### Overall survival

The 3-year OS in the nonR-RIC and R-RIC groups was 56% (95%CI 53–59) and 64% (95%CI 59–68), respectively, *p* = 0.01 (Table 2). There was a significant center effect noted for OS (*p* = 0.005). On multivariate analysis (Table 3), after adjusting for the center effect, R-RIC was not associated with a significantly reduced risk of mortality (RR = 0.84, 95%CI = 0.69–1.02, *p* = 0.08) relative to nonR-RIC allo-HCT (Fig. 1e). Other factors independently associated with mortality risk are shown in the Additional file 1: Table S2.

### Rituximab and disease histology

The interaction between the main effect (i.e., R-RIC vs. RIC) and disease histology (DLBCL, FL, MCL, and MZL) was checked for each outcome. It was not significant for grade II–IV acute GVHD (*p* = 0.18), grade III–IV acute GVHD (*p* = 0.64), chronic GVHD (*p* = 0.99), relapse (*p* = 0.96), NRM (*p* = 0.70), PFS (*p* = 0.84), and OS (*p* = 0.53) (For details of histology-specific multivariate models please refer to page 10 of Additional file 1: Tables S3-S5). The subset analysis for chemorefractory patients at the time of HCT is given in Additional file 1: Table S6.

### Prior autologous transplant

On multivariate analysis, independent of the type of RIC transplant (R or nonR), prior auto-HCT was associated with higher NRM (HR = 1.38, 95%CI 1.07–1.78, *p* = 0.01), inferior PFS (HR = 1.28, 95%CI 1.08–1.51, *p* = 0.004), and OS (HR = 1.22, 95%CI 1.02–1.46, *p* = 0.02) (Additional file 1: Table S2).

### Pre-transplant rituximab

Among nonR-RIC and R-RIC groups, 149 and 89 cases on the CRF track had details of pre-transplant treatments available. Among this subset, 54% (*n* = 80) of nonR-RIC and 64% (*n* = 57) of R-RIC patients received rituximab as a part of last line therapy prior to transplantation (*p* = 0.12).

### Causes of death

At last follow-up, 43% (*n* = 444) of nonR-RIC and 37% (*n* = 139) of R-RIC transplant recipients had died (Additional file 1: Table-S6). The most common cause of death in both cohorts was recurrent/progressive lymphoma; 18% (*n* = 186) and 14% (*n* = 52) in the nonR-RIC and R-RIC groups, respectively. GVHD was the cause of death in 4% (*n* = 36) and 6% (*n* = 21) of recipients of nonR-RIC and R-RIC transplant, respectively. Infectious complications led to the death of 3% (*n* = 29) in nonR-RIC group and 3% (*n* = 13) in R-RIC group. Among the nonR-RIC and R-RIC groups, 157 and 89 cases on the CRF track had detailed post-transplant infectious disease data available. Among this subset, 26% (*n* = 41) of nonR-RIC and 20% (*n* = 18) of R-RIC patients had CMV reactivation (*p* = 0.20) and 1% (*n* = 2) of nonR-RIC and 2% (*n* = 2) of R-RIC patients had EBV reactivation (*p* = 0.60). Neither of the groups (R-RIC or nonR-RIC) had any HBV reactivation cases.

### Subset analysis

Among the 381 patients receiving Flu/Bu-based RIC, only 21 patients (5%) received rituximab-based conditioning. Results of multivariate analysis after excluding the patients receiving Flu/Bu-based conditioning are shown in Table 4. These results were generally in line with multivariate analysis of overall study population and showed that relative to non-R-RIC group, the R-RIC group was associated with no difference in grade III–IV acute GVHD (RR = 0.89, 95%CI = 0.59–1.34, *p* = 0.58), chronic GVHD (RR = 1.07, 95%CI = 0.87–1.30, *p* = 0.51), NRM (RR = 0.79, 95%CI = 0.57–1.08, *p* = 0.14), or progression/relapse (RR = 0.82, 95%CI = 0.64–1.06, *p* = 0.13), and the R-RIC significantly improved PFS (RR = 0.002, 95%CI = 0.60–0.89, *p* = 0.002). However, unlike the overall multivariate model, this subgroup analysis showed a significantly reduced risk of mortality with R-RIC (RR = 0.76, 95%CI = 0.60–0.96, *p* = 0.02), relative to the nonR-RIC cohort.

**Table 4:** Multivariate analysis results (excluding Fludarabine/Busulfan-based conditioning regimens)

	Number	Relative risk	95%CI lower limit	95%CI upper limit	*p* value
Acute GVHD (grades 3–4)^a^					
Non-rituximab RIC	647	1			0.58
Rituximab-containing RIC	356	0.89	0.59	1.34	
Chronic GVHD					
Non-rituximab RIC	635	1			0.51
Rituximab-containing RIC	349	1.07	0..87	1.30	
Non-relapse mortality					
Non-rituximab RIC	638	1			0.14
Rituximab-containing RIC	346	0.79	0.57	1.08	
Progression/relapse					
Non-rituximab RIC	638	1			0.13
Rituximab-containing RIC	346	0.82	0.64	1.06	
PFS					
Non-rituximab RIC	638	1			0.002
Rituximab-containing RIC	346	0.73	0.60	0.89	
Mortality					
Non-rituximab RIC	662	1			0.02
Rituximab-containing RIC	358	0.76	0.60	0.96	

*Abbreviations*: *GVHD* graft-versus-host disease, *RIC* reduced-intensity conditioning

^a^Acute GVHD models used logistic regression

### Effect of cumulative rituximab dose in conditioning

The cumulative (or total) dose of rituximab administered with allo-HCT conditioning varies significantly across different centers. To assess the impact of cumulative rituximab dose on HCT outcomes, we divided the R-RIC cohort patients into three groups based on the cumulative rituximab dose received during RIC—<1000 mg/m^2^ (low; *n* = 214), 1000–1999 mg/m^2^ (intermediate; *n* = 113), and 2000–3375 mg/m^2^ (high; *n* = 47). On multivariate analysis (Table 5), there was a reduced risk of chronic GVHD with the intermediate rituximab dose (RR = 0.64; 95%CI 0.46–0.90, *p* = 0.01) relative to the low dose rituximab group. High dose rituximab was associated with a reduced risk of NRM (RR = 0.18; 95%CI 0.04–0.75; *p* = 0.02) and overall mortality (RR = 0.43; 95%CI 0.21–0.90; *p* = 0.02), relative to the low dose group.

**Table 5:** Effect of cumulative rituximab dose on outcomes

	Number	Relative risk	95%CI lower limit	95%CI upper limit	*p* value	Overall *p* value
Chronic GVHD						
Rituximab dose (mg/m^2^)						
<1000	208	1				0.03
1000–1999	110	0.64	0.46	0.90	0.01	
2000–3375	46	0.92	0.62	1.36	0.67	
Non-relapse mortality						
Rituximab dose (mg/m^2^)						
<1000	208	1				0.06
1000–1999	109	0.90	0.54	1.51	0.70	
2000–3375	45	0.18	0.04	0.75	0.02	
Progression/relapse						
Rituximab dose (mg/m^2^)						
<1000	208	1				0.90
1000–1999	109	1.01	0.63	1.61	0.97	
2000–3375	45	0.85	0.40	1.78	0.66	
PFS						
Rituximab dose (mg/m^2^)						
<1000	208	1				0.21
1000–1999	109	0.99	0.70	1.39	0.94	
2000–3375	45	0.56	0.30	1.07	0.08	
Mortality						
Rituximab dose (mg/m^2^)						
<1000	214	1				0.052
1000–1999	113	1.10	0.76	1.59	0.63	
2000–3375	47	0.43	0.21	0.90	0.02	

*Abbreviations*: *GVHD* graft-versus-host disease

## Discussion

Prospective randomized studies comparing the outcomes of R-RIC and nonR-RIC in B cell NHL have not been performed. Here, we performed a registry analysis of B cell NHL patients receiving either rituximab-based or nonR-RIC regimens for allo-HCT, and make several important observations. First rates of hematopoietic recovery, relapse risk, and NRM were comparable between R-RIC and nonR-RIC allo-HCT. Second, R-RIC was not associated with a higher risk of either grade II–IV or grade III–IV acute GVHD and chronic GVHD. Third, there was a significantly superior PFS with R-RIC regimens, but in the overall study population, OS was similar. Finally, in the subset analysis, there was a survival benefit in favor of R-RIC regimens (after excluding Flu/Bu-based conditioning regimens) and a higher cumulative rituximab dose was associated with a reduced risk of NRM and improved survival.

In our study, R-RIC group was not associated with a higher risk of grade II–IV or grade III–IV acute GVHD compared to the nonR-RIC cohort. Our analysis also did not show any difference in the risk of chronic GVHD between the two groups. Previous studies using R-RIC have generally shown the rates of chronic GVHD at 1 year to be around 50–60% (Additional file 1: Table S7) [4–6, 8]. Notably, some retrospective and one prospective phase II study using a prolonged rituximab administration schedule post allo-HCT have suggested reduced risk of chronic GVHD [7, 18, 19]. However, a randomized trial did not show any reduction in chronic GVHD with post allo-HCT administration of rituximab. Of note, all patients in that study received myeloablative conditioning regimens [20]. A recent single-center retrospective study comparing fludarabine, cyclophosphamide, and rituximab (FCR) RIC to Flu/Bu (without rituximab) [21] reported lower rates of chronic GVHD and improved OS with R-RIC. However, in that study, all the patients in the FCR group received tacrolimus/methotrexate as GVHD prophylaxis, while those in the Flu/Bu group received tacrolimus/mycophenolate mofetil as GVHD prophylaxis that confounds the assessment of rituximab’s impact on the rates of chronic GVHD and survival.

Single-arm studies have shown excellent survival outcomes with R-RIC in B cell NHL [4, 8, 21]. Additional file 1: Table S7 summarizes the studies that looked at the addition of rituximab to RIC backbone in an effort to improve the outcomes. The current study is the first multicenter validation of these results, wherein there was a significant improvement in PFS in R-RIC group compared to the nonR-RIC group. Of note, in our study, the OS benefit emerged in the subset analysis excluding Flu/Bu patients. The fact that Flu/Bu was consistently associated with the lowest risk of NRM and improved PFS and OS (Additional file 1: Table S2) and that only 5% of Flu/Bu patients got R-RIC in our study prompted us to perform a subgroup multivariate analysis after excluding this conditioning approach. Whether the survival benefit that emerged in the non-Flu/Bu type R-RIC regimens also exists for Flu/Bu-based regimens requires further investigation.

In the recently reported BMT CTN 0701 study, OS was significantly better among patients who achieved a higher serum concentration of rituximab versus those with a lower serum concentration at day 28 [8]. The association between rituximab dose and serum rituximab concentration is well known [22]. In line with the observations made in BMT CTN 0701, in the current analysis, we observed that the patients who received a higher cumulative rituximab dose (a possible surrogate for higher serum concentrations) had better OS. We advise caution in interpreting the data of reduced mortality and NRM with higher cumulative rituximab dose in our study, given the small sample size (*n* = 45), the observed wide confidence intervals, and the non-significant overall *p* value of the model (Table 5). Limited data suggest that rituximab in conditioning or its early application post allo-HCT might be associated with prolonged life-threatening cytopenias [23]. In our study, rituximab use was not associated with delayed neutrophil recovery or fatal infections. Mortality due to GVHD was also comparable between the two groups. Four percent of deaths in the nonR-RIC group were due to GVHD as opposed to 6% in the R-RIC group (Additional file 1: Table S6).

Unfortunately, one of the limitations of the registry analysis is that we only capture the cumulative rituximab dose (at conditioning) and do not capture the exact dosing schedule. Additionally, only a small number of patients (*n* = 36) received post-transplant rituximab, thereby limiting the ability to assess the impact of post-transplant rituximab on the outcomes.

## Conclusions

Our analysis is the largest comparative study evaluating outcomes of R-RIC versus nonR-RIC in B cell NHL patients. Although there was an increase in the risk of grade II–IV acute GVHD with R-RIC regimens, there was no increase in the risk of grade III–IV acute GVHD or chronic GVHD. There was a significant improvement in PFS with R-RIC allo-HCT in our study. Survival benefit was noted in the R-RIC group after exclusion of Flu/Bu conditioning regimen. Additionally, higher cumulative rituximab dose was associated with significantly improved survival. In the absence of randomized data, our results suggest that R-RIC should be considered as the preferred conditioning regimen for B cell lymphomas. Our encouraging data warrants confirmation in a randomized trial setting.

## Additional file

Additional file 1:
**Table S1.** Variables tested in Cox proportional hazards regression models. **Table S2.** Complete multivariate analysis results. **Table S3.** Multivariate analysis results for follicular lymphoma. **Table S4.** Multivariate analysis results for mantle cell lymphoma. **Table S5.** Multivariate analysis results for diffuse large B cell lymphoma. **Table S6.** Univariate analysis—chemorefractory patients. **Table S7.** Causes of death. **Table S8.** Studies incorporating rituximab to allo-HCT conditioning regimens in B cell NHL (DOCX 81 kb)

## Abbreviations

Allo-HCT: Allogeneic hematopoietic cell transplantation
B-NHL: B cell non-Hodgkin lymphoma
CNI: Calcineurin inhibitor
CR: Complete remission
CRF: Comprehensive Report Form
DLBCL: Diffuse large B cell lymphoma
FCR: Fludarabine, cyclophosphamide, and rituximab
FL: Follicular lymphoma
Flu/Bu: Fludarabine, busulfan
GVHD: Graft-versus-host disease
MCL: Mantle cell lymphoma
MZL: Marginal zone lymphoma
NonR-RIC: Rituximab-containing reduced-intensity conditioning
NRM: Non-relapse mortality
OS: Overall survival
PFS: Progression-free survival
PR: Partial remission
RR: Relative risk
R-RIC: Rituximab-containing reduced-intensity conditioning
TBI: Total body irradiation
TED: Transplant Essential Data

## References

[1] Pasquini MC, Zhu X. Current uses and outcomes of hematopoietic stem cell transplantation: CIBMTR Summary Slides. 2015. http://www.cibmtr.org.

[2] Hamadani M, Saber W, Ahn KW (2013). Impact of pre-transplantation conditioning regimens on outcomes of allogeneic transplantation for chemotherapy-unresponsive diffuse large B cell lymphoma and grade III follicular lymphoma. Biol Blood Marrow Transplant.

[3] Hari P, Carreras J, Zhang MJ (2008). Allogeneic transplants in follicular lymphoma: higher risk of disease progression after reduced-intensity compared to myeloablative conditioning. Biol Blood Marrow Transplant.

[4] Khouri IF, McLaughlin P, Saliba RM (2008). Eight-year experience with allogeneic stem cell transplantation for relapsed follicular lymphoma after nonmyeloablative conditioning with fludarabine, cyclophosphamide, and rituximab. Blood.

[5] Pidala J, Roman-Diaz J, Kim J (2011). Targeted IV busulfan and fludarabine followed by post-allogeneic hematopoietic cell transplantation rituximab demonstrate encouraging activity in CD20+ lymphoid malignancies without increased risk of infectious complications. Int J Hematol.

[6] Kharfan-Dabaja MA, Anasetti C, Fernandez HF (2013). Phase II study of CD4+-guided pentostatin lymphodepletion and pharmacokinetically targeted busulfan as conditioning for hematopoietic cell allografting. Biol Blood Marrow Transplant.

[7] Sauter CS, Jn B, Lechner L (2014). A phase II study of a nonmyeloablative allogeneic stem cell transplant with peritransplant rituximab in patients with B cell lymphoid malignancies: favorably durable event-free survival in chemosensitive patients. Biol Blood Marrow Transplant.

[8] Laport GG, Wu J, Logan B (2016). Reduced-intensity conditioning with fludarabine, cyclophosphamide, and high-dose rituximab for allogeneic hematopoietic cell transplantation for follicular lymphoma: a phase two multicenter trial from the blood and marrow transplant clinical trials network. Biol Blood Marrow Transplant.

[9] Bacigalupo A, Ballen K, Rizzo D (2009). Defining the intensity of conditioning regimens: working definitions. Biol Blood Marrow Transplant.

[10] Cheson BD, Pfistner B, Juweid ME (2007). Revised response criteria for malignant lymphoma. J Clin Oncol.

[11] Armand P, Kim H, Logan B (2014). Validation and refinement of the disease risk index for allogeneic stem cell transplantation. Blood.

[12] Przepiorka D, Weisdorf D, Martin P (1995). 1994 Consensus conference on acute GVHD grading. Bone Marrow Transplant.

[13] Shulman H, Sullivan K, Weiden P (1980). Chronic graft-versus-host syndrome in man. A long-term clinicopathologic study of 20 Seattle patients. Am J Med.

[14] Zhang X, Loberiza FR, Klein JP (2007). A SAS macro for estimation of direct adjusted survival curves based on a stratified Cox regression model. Comput Methods Programs Biomed.

[15] Zhang X, Zhang M (2011). SAS macros for estimation of direct adjusted cumulative incidence curves under proportional sub-distribution hazards models. Comput Methods Programs Biomed.

[16] Commenges D, Andersen PK (1995). Score test of homogeneity for survival data. Lifetime Data Anal.

[17] Lee EW, Wei LJ, Amato D (1992). Cox-type regression analysis for large numbers of small groups of correlated failure time observations.

[18] Arai S, Sahaf B, Narasimhan B (2012). Prophylactic rituximab after allogeneic transplantation decreases B-cell alloimmunity with low chronic GVHD incidence. Blood.

[19] Cutler C, Kim HT, Bindra B (2013). Rituximab prophylaxis prevents corticosteroid-requiring chronic GVHD after allogeneic peripheral blood stem cell transplantation: results of a phase 2 trial. Blood.

[20] Glass B, Hasenkamp J, Wulf G (2014). Rituximab after lymphoma-directed conditioning and allogeneic stem-cell transplantation for relapsed and refractory aggressive non-Hodgkin lymphoma (DSHNHL R3): an open label, randomized phase 2 trial. Lancet Oncol.

[21] Kennedy VE, Savani BN, Greer JP (2016). Reduced-intensity conditioning with fludarabine, cyclophosphamide, and rituximab is associated with improved outcomes compared with fludarabine and busulfan after allogeneic stem cell transplantation for B-cell malignancies. Biol Blood Marrow Transplant.

[22] Tobinai K, Kobayashi Y, Narabayashi M (1998). Feasibility and pharmacokinetic study of a chimeric anti-CD20 monoclonal antibody (IDEC-C2B8, rituximab) in relapsed B-cell lymphoma. The IDEC-C2B8 Study Group. Ann Oncol.

[23] McIver Z, Stephens N, Grim A (2010). Rituximab administration within 6 months of T cell-depleted allogeneic SCT is associated with prolonged life-threatening cytopenias. Biol Blood Marrow Transplant.

