# Supplementary material for: Rituximab-containing reduced-intensity conditioning improves progression-free survival following allogeneic transplantation in B cell non-Hodgkin lymphoma
Source: J Hematol Oncol. 2017 Jun 12;10:117. doi: 10.1186/s13045-017-0487-y (PMC5469142; doi:10.1186/s13045-017-0487-y)
Supplement: Additional file 1: — Table S1. Variables tested in Cox proportional hazards regression models. Table S2. Complete multivariate analysis results. Table S3. Multivariate analysis results for follicular lymphoma. Table S4. Multivariate analysis results for mantle cell lymphoma. Table S5. Multivariate analysis results for diffuse large B cell lymphoma. Table S6. Univariate analysis—chemorefractory patients. Table S7. Causes of death. Table S8. Studies incorporating rituximab to allo-HCT conditioning regimens in B cell NHL (DOCX 81 kb) [file 13045_2017_487_MOESM1_ESM.docx]

**Additional file**

**Table of Contents 1**

**SUPPLEMENTARY TABLES**  **2-8**

Table S1 2

Table S2 3

**RITUXIMAB & DISEASE HISTOLOGY 9-11**

**Follicular lymphoma** Table S3 9

**MCL** Table S4 10

**DLBCL** Table S5 11

**Univariate Analysis of Chemorefractory Patients:** Table S6 12

**Causes of Death:** Table S7 13

**Summary of Published Data:** Table S8 14

**Details of GVHD prophylaxis:** Table S9 15

**Table S1. Variables tested in Cox proportional hazards regression models**

**Main Effect:**

- Rituximab containing conditioning vs. non Rituximab-containing conditioning

**Patient related:**

- Age at transplant continuous to find appropriate cut point for survival model
- Race: Caucasians vs. others (including AA) vs. missing
- Karnofsky performance status at transplant: 90-100% vs. <90 vs. missing
- Sorror Co-morbidity index : 0 vs. 1-2 vs. 3 or more

**Disease related:**

- Disease subtype: FL vs DLBCL vs MCL vs MZL
- Time from diagnosis to HCT: <1 year vs. ≥1 year
- Chemosensitivity at alloHCT: CR vs. PR vs. chemoresistant vs. untreated/missing
- Prior autologous transplant: no vs yes

**Transplant related:**

- Year of transplant: continuous
- ATG in conditioning regimen: Yes vs. No
- Donor type: HLA-identical sibling vs. URD
- Year of HCT: Continuous to find the appropriate cut point for the survival model
- Conditioning regimens: Flu/Bu/TBI-based vs. Flu/Cy/TBI-based vs. Flu/Mel/TBI-based vs. BEAM-like vs. 2GyTBI+/-Flu based only
- GVHD prophylaxis: calcineurin inhibitors + MTX ± others (excluding MMF/Siro) vs. calcineurin inhibitors + MMF ± others (excluding Siro) vs. calcineurin inhibitors + Sirolimus ± others vs. calcineurin inhibitors ± others (excluding MTX/MMF/Siro)
- Donor/Recipient Sex: female-female vs. female-male vs. male-male vs. male-female
- Donor/Recipient CMV status : -/+ vs. others

**Table S2. Complete Multivariate Analysis Results**

|  | **Number** | **Odds Ratio** | **95% CI**  **Lower Limit** | **95% CI**  **Upper Limit** | **P-value** | **Overall**  **p-value** |
| --- | --- | --- | --- | --- | --- | --- |
| **Acute GVHD (Grades 2-4)*** |  |  |  |  |  |  |
| **Main Effect** |  |  |  |  |  |  |
| Non-Rituximab RIC | 1004 | 1 |  |  |  | 0.43 |
| Rituximab-containing RIC | 377 | 1.14 | 0.83 | 1.56 | 0.43 |  |
| **Disease subtype** |  |  |  |  |  |  |
| FL | 405 | 1 |  |  |  | 0.007 |
| DLBCL | 568 | 0.86 | 0.65 | 1.15 | 0.32 |  |
| MCL | 374 | 1.43 | 1.05 | 1.95 | 0.02 |  |
| MZL | 34 | 1.09 | 0.50 | 2.37 | 0.82 |  |
| **Chemosensitivity** |  |  |  |  |  |  |
| CR | 598 | 1 |  |  |  | 0.002 |
| PR | 579 | 1.26 | 0.97 | 1.63 | 0.08 |  |
| Chemoresistant | 183 | 1.87 | 1.29 | 2.71 | 0.0009 |  |
| Untreated/Missing | 21 | 2.75 | 1.09 | 6.94 | 0.03 |  |
| **Donor type** |  |  |  |  |  |  |
| HLA identical sibling | 743 | 1 |  |  |  | 0.0002 |
| URD 8/8 | 638 | 1.61 | 1.27 | 2.05 | 0.0002 |  |
| **GVHD prophylaxis** |  |  |  |  |  |  |
| CNI +MTX (+/- others) | 593 | 1 |  |  |  | 0.004 |
| CNI + MMF (+/- others) | 486 | 1.28 | 0.93 | 1.76 | 0.13 |  |
| CNI + Sirolimus (+/- others) | 273 | 0.70 | 0.47 | 1.04 | 0.08 |  |
| CNI alone | 29 | 0.23 | 0.06 | 0.80 | 0.02 |  |
| **Acute GVHD (Grades 3-4)*** |  |  |  |  |  |  |
| **Main Effect** |  |  |  |  |  |  |
| Non-Rituximab RIC | 1004 | 1 |  |  |  | 0.54 |
| Rituximab-containing RIC | 377 | 1.16 | 0.72 | 1.89 | 0.54 |  |
| **Disease subtype** |  |  |  |  |  |  |
| FL | 405 | 1 |  |  |  | 0.004 |
| DLBCL | 568 | 0.99 | 0.64 | 1.52 | 0.96 |  |
| MCL | 374 | 1.88 | 1.23 | 2.87 | 0.004 |  |
| MZL | 34 | 1.50 | 0.56 | 4.02 | 0.42 |  |
| **Chemosensitivity** |  |  |  |  |  |  |
| CR | 598 | 1 |  |  |  | 0.02 |
| PR | 579 | 1.54 | 1.07 | 2.23 | 0.02 |  |
| Chemoresistant | 183 | 1.77 | 1.06 | 2.96 | 0.03 |  |
| Missing/Untreated | 21 | 3.22 | 1.09 | 9.50 | 0.03 |  |
| **Donor type** |  |  |  |  |  |  |
| HLA identical sibling | 743 | 1 |  |  |  | 0.0008 |
| **Table S2 continued** | **Number** | **Odds Ratio** | **95% CI**  **Lower Limit** | **95% CI**  **Upper Limit** | **P-value** | **Overall**  **p-value** |
| URD 8/8 | 638 | 1.77 | 1.27 | 2.48 | 0.0008 |  |
| **Conditioning regimen** |  |  |  |  |  |  |
| Flu/Bu ± TBI | 378 | 1 |  |  |  | 0.02 |
| Flu/Cy ± TBI | 361 | 1.56 | 0.82 | 2.97 | 0.18 |  |
| Flu/Mel ± TBI | 320 | 2.11 | 1.19 | 3.75 | 0.01 |  |
| BEAM and similar | 94 | 3.19 | 1.45 | 6.90 | 0.003 |  |
| 2GyTBI ± Flu | 228 | 2.19 | 1.14 | 4.19 | 0.02 |  |
| **Chronic GVHD** |  |  |  |  |  |  |
| **Main Effect** |  |  |  |  |  |  |
| Non-Rituximab RIC | 982 | 1 |  |  |  | 0.22 |
| Rituximab-containing RIC | 369 | 1.15 | 0.92 | 1.46 | 0.22 |  |
| **Donor type** |  |  |  |  |  |  |
| HLA identical sibling | 727 | 1 |  |  |  | 0.004 |
| URD 8/8 | 624 | 1.25 | 1.07 | 1.45 | 0.004 |  |
| **GVHD prophylaxis (<= 8 months)** |  |  |  |  |  |  |
| CNI + MTX (+/- others) | 582 | 1 |  |  |  | 0.03 |
| CNI + MMF (+/-others) | 480 | 1.49 | 1.09 | 2.04 | 0.01 |  |
| CNI + Sirolimus (+/- others) | 260 | 0.99 | 0.75 | 1.31 | 0.96 |  |
| CNI +/- others | 29 | 0.61 | 0.23 | 1.64 | 0.33 |  |
| **GVHD prophylaxis (> 8 months)** |  |  |  |  |  |  |
| CNI +MTX (+/- others) | 310 | 1 |  |  |  | <.0001 |
| CNI MTX (+/- others) | 183 | 1.33 | 0.99 | 1.79 | 0.05 |  |
| CNI + Sirolimus (+/- others) | 141 | 1.80 | 1.41 | 2.31 | <.0001 |  |
| CNI alone | 20 | 0.44 | 0.15 | 1.28 | 0.13 |  |
| **Conditioning regimen** |  |  |  |  |  |  |
| Flu/Bu ± TBI | 367 | 1 |  |  |  | 0.0003 |
| Flu/Cy ± TBI | 350 | 0.62 | 0.45 | 0.84 | 0.003 |  |
| Flu/Mel ± TBI | 312 | 0.95 | 0.75 | 1.21 | 0.69 |  |
| BEAM and similar | 93 | 1.22 | 0.91 | 1.62 | 0.19 |  |
| 2GyTBI ± Flu | 229 | 0.88 | 0.61 | 1.28 | 0.52 |  |
| **ATG** |  |  |  |  |  |  |
| No | 1092 | 1 |  |  |  | <.0001 |
| Yes | 259 | 0.46 | 0.36 | 0.58 | <.0001 |  |
| **Relapse/Progression** |  |  |  |  |  |  |
| **Main Effect** |  |  |  |  |  |  |
| Non-Rituximab RIC | 988 | 1 |  |  |  | 0.055 |
| Rituximab-containing RIC | 367 | 0.79 | 0.63 | 1.005 | 0.055 |  |
| **Age** |  |  |  |  |  |  |
| 18-40 | 84 | 1 |  |  |  | 0.009 |
| **Table S2 continued** | **Number** | **Hazard Ratio** | **95% CI**  **Lower Limit** | **95% CI**  **Upper Limit** | **P-value** | **Overall**  **p-value** |
| 41-50 | 210 | 1.62 | 1.03 | 2.55 | 0.04 |  |
| 51-60 | 565 | 1.05 | 0.68 | 1.60 | 0.84 |  |
| >60 | 496 | 1.34 | 0.88 | 2.06 | 0.17 |  |
| **Disease subtype** |  |  |  |  |  |  |
| FL | 389 | 1 |  |  |  | <.0001 |
| DLBCL | 564 | 3.35 | 2.51 | 4.48 | <.0001 |  |
| MCL | 370 | 2.88 | 2.10 | 3.95 | <.0001 |  |
| MZL | 32 | 1.5 | 0.68 | 3.29 | 0.31 |  |
| **Chemosensitivity** |  |  |  |  |  |  |
| CR | 596 | 1 |  |  |  | <.0001 |
| PR | 566 | 1.84 | 1.47 | 2.31 | <.0001 |  |
| Chemoresistant | 173 | 3.61 | 2.74 | 4.75 | <.0001 |  |
| Missing/Untreated | 20 | 1.41 | 0.52 | 3.84 | 0.50 |  |
| **Donor type** |  |  |  |  |  |  |
| HLA identical sibling | 730 | 1 |  |  |  | 0.001 |
| URD 8/8 | 625 | 0.72 | 0.58 | 0.88 | 0.001 |  |
| **GVHD prophylaxis** |  |  |  |  |  |  |
| CNI +MTX (+/- others) | 578 | 1 |  |  |  | 0.003 |
| CNI + MMF (+/- others) | 475 | 0.91 | 0.73 | 1.13 | 0.40 |  |
| CNI + Sirolimus (+/- others) | 272 | 0.64 | 0.49 | 0.85 | 0.002 |  |
| CNI alone | 30 | 1.61 | 0.91 | 2.85 | 0.10 |  |
| **Sex match** |  |  |  |  |  |  |
| FF | 583 | 1 |  |  |  | 0.04 |
| FM | 299 | 1.03 | 0.79 | 1.33 | 0.83 |  |
| MM | 303 | 0.69 | 0.53 | 0.91 | 0.008 |  |
| MF | 170 | 1.01 | 0.74 | 1.36 | 0.97 |  |
| **Non Relapse Mortality** |  |  |  |  |  |  |
| **Main Effect** |  |  |  |  |  |  |
| Non-Rituximab RIC | 988 | 1 |  |  |  | 0.51 |
| Rituximab-containing RIC | 367 | 0.90 | 0.67 | 1.22 | 0.51 |  |
| **Age** |  |  |  |  |  |  |
| 18-40 | 84 | 1 |  |  |  | 0.0001 |
| 41-50 | 210 | 1.41 | 0.68 | 1.41 | 0.36 |  |
| 51-60 | 565 | 1.58 | 0.83 | 1.58 | 0.16 |  |
| >60 | 496 | 2.22 | 1.15 | 2.22 | 0.02 |  |
| **KPS** |  |  |  |  |  |  |
| >=90% | 839 | 1 |  |  |  | 0.002 |
| <90% | 481 | 1.43 | 1.16 | 1.75 | 0.0007 |  |
| Missing | 35 | 1.41 | 0.69 | 2.89 | 0.35 |  |
| **Table S2 continued** | **Number** | **Hazard Ratio** | **95% CI**  **Lower Limit** | **95% CI**  **Upper Limit** | **P-value** | **Overall**  **p-value** |
| **Sorror Co-morbidity index** |  |  |  |  |  |  |
| 0 | 455 | 1 |  |  |  | 0.003 |
| 1-2 | 407 | 1.19 | 0.90 | 1.57 | 0.22 |  |
| 3 | 429 | 1.72 | 1.28 | 2.31 | 0.0003 |  |
| Missing | 64 | 1.49 | 0.65 | 3.43 | 0.34 |  |
| **Donor type** |  |  |  |  |  |  |
| HLA identical sibling | 730 | 1 |  |  |  | <.0001 |
| URD 8/8 | 625 | 1.65 | 1.29 | 2.12 | <.0001 |  |
| **Conditioning regimen** |  |  |  |  |  |  |
| Flu/Bu ± TBI | 371 | 1 |  |  |  | <.0001 |
| Flu/Cy ± TBI | 356 | 1.12 | 0.64 | 1.94 | 0.69 |  |
| Flu/Mel ± TBI | 313 | 2.48 | 1.57 | 3.90 | <.0001 |  |
| BEAM and similar | 89 | 3.87 | 2.34 | 6.40 | <.0001 |  |
| 2GyTBI ± Flu | 226 | 1.83 | 1.19 | 2.82 | 0.006 |  |
| **Prior autologous transplant** |  |  |  |  |  |  |
| No | 830 | 1 |  |  |  | 0.005 |
| Yes | 525 | 1.38 | 1.1 | 1.73 | 0.005 |  |
| **Progression free survival** |  |  |  |  |  |  |
| **Main Effect** |  |  |  |  |  |  |
| Non-Rituximab RIC | 988 | 1 |  |  |  | 0.006 |
| Rituximab-containing RIC | 367 | 0.76 | 0.62 | 0.92 | 0.006 |  |
| **Age** |  |  |  |  |  |  |
| 18-40 | 84 | 1 |  |  |  | 0.0007 |
| 41-50 | 210 | 1.59 | 1.09 | 2.31 | 0.02 |  |
| 51-60 | 565 | 1.21 | 0.85 | 1.71 | 0.29 |  |
| >60 | 496 | 1.62 | 1.14 | 2.29 | 0.007 |  |
| **KPS** |  |  |  |  |  |  |
| >=90% | 839 | 1 |  |  |  | 0.01 |
| <90% | 481 | 1.27 | 1.08 | 1.49 | 0.004 |  |
| Missing | 35 | 1.21 | 0.77 | 1.89 | 0.40 |  |
| **Sorror Co-morbidity index** |  |  |  |  |  |  |
| 0 | 455 | 1 |  |  |  | 0.003 |
| 1-2 | 407 | 1.14 | 0.94 | 1.40 | 0.18 |  |
| 3 | 429 | 1.41 | 1.16 | 1.71 | 0.0005 |  |
| Missing | 64 | 0.97 | 0.64 | 1.49 | 0.91 |  |
| **Disease subtype** |  |  |  |  |  |  |
| FL | 389 | 1 |  |  |  | <.0001 |
| DLBCL | 564 | 2.11 | 1.69 | 2.62 | <.0001 |  |
| MCL | 370 | 2.05 | 1.63 | 2.58 | <.0001 |  |
| **Table S2 continued** | **Number** | **Hazard Ratio** | **95% CI**  **Lower Limit** | **95% CI**  **Upper Limit** | **P-value** | **Overall**  **p-value** |
| MZL | 32 | 1.33 | 0.76 | 2.32 | 0.32 |  |
| **Chemosensitivity** |  |  |  |  |  |  |
| CR | 596 | 1 |  |  |  | <.0001 |
| PR | 566 | 1.61 | 1.35 | 1.91 | <.0001 |  |
| Chemoresistant | 173 | 2.67 | 2.14 | 3.35 | <.0001 |  |
| Missing/Untreated | 20 | 1.95 | 1.08 | 3.53 | 0.03 |  |
| **Conditioning regimen** |  |  |  |  |  |  |
| Flu/Bu ± TBI | 371 | 1 |  |  |  | 0.0007 |
| Flu/Cy ± TBI | 356 | 1.30 | 1.01 | 1.66 | 0.04 |  |
| Flu/Mel ± TBI | 313 | 1.51 | 1.22 | 1.87 | 0.0002 |  |
| BEAM and similar | 89 | 1.77 | 1.27 | 2.47 | 0.0007 |  |
| 2GyTBI ± Flu | 226 | 1.15 | 0.88 | 1.49 | 0.29 |  |
| **GVHD prophylaxis** |  |  |  |  |  |  |
| CNI +MTX (+/- others) | 578 | 1 |  |  |  | 0.002 |
| CNI + MMF (+/- others) | 475 | 1.06 | 0.87 | 1.29 | 0.57 |  |
| CNI + Sirolimus (+/- others) | 272 | 0.71 | 0.57 | 0.89 | 0.003 |  |
| CNI alone | 30 | 1.48 | 0.89 | 2.45 | 0.13 |  |
| **Sex match** |  |  |  |  |  |  |
| FF | 583 | 1 |  |  |  | 0.008 |
| FM | 299 | 1.15 | 0.94 | 1.39 | 0.16 |  |
| MM | 303 | 0.77 | 0.62 | 0.94 | 0.01 |  |
| MF | 170 | 1.009 | 0.79 | 1.29 | 0.94 |  |
| **Prior autologous transplant** |  |  |  |  |  |  |
| No | 830 | 1 |  |  |  | 0.004 |
| Yes | 525 | 1.28 | 1.08 | 1.51 | 0.004 |  |
| **Mortality (inverse of OS)** |  |  |  |  |  |  |
| **Main Effect** |  |  |  |  |  |  |
| Non-Rituximab RIC | 1022 | 1 |  |  |  | 0.08 |
| Rituximab-containing RIC | 379 | 0.84 | 0.69 | 1.02 | 0.08 |  |
| **Age** |  |  |  |  |  |  |
| 18-40 | 86 | 1 |  |  |  | <0.0001 |
| 41-50 | 214 | 1.33 | 0.90 | 1.99 | 0.15 |  |
| 51-60 | 592 | 1.34 | 0.93 | 1.92 | 0.11 |  |
| >60 | 509 | 1.82 | 1.26 | 2.62 | 0.001 |  |
| **KPS** |  |  |  |  |  |  |
| >=90% | 857 | 1 |  |  |  | <.0001 |
| <90% | 506 | 1.47 | 1.25 | 1.72 | <.0001 |  |
| Missing | 38 | 1.18 | 0.84 | 1.66 | 0.55 |  |
| **Sorror Co-morbidity index** |  |  |  |  |  |  |
| **Table S2 continued** | **Number** | **Hazard Ratio** | **95% CI**  **Lower Limit** | **95% CI**  **Upper Limit** | **P-value** | **Overall**  **p-value** |
| 0 | 472 | 1 |  |  |  | 0.02 |
| 1-2 | 416 | 1.05 | 0.87 | 1.26 | 0.61 |  |
| 3 | 446 | 1.42 | 1.13 | 1.77 | 0.002 |  |
| Missing | 67 | 1.15 | 0.78 | 1.68 | 0.48 |  |
| **Disease subtype** |  |  |  |  |  |  |
| FL | 410 | 1 |  |  |  | <.0001 |
| DLBCL | 576 | 1.87 | 1.49 | 2.35 | <.0001 |  |
| MCL | 380 | 1.7 | 1.33 | 2.16 | <.0001 |  |
| MZL | 35 | 1.53 | 1.01 | 2.31 | 0.04 |  |
| **Chemosensitivity** |  |  |  |  |  |  |
| CR | 606 | 1 |  |  |  | <.0001 |
| PR | 590 | 1.51 | 1.22 | 1.86 | 0.0001 |  |
| Chemoresistant | 184 | 2.17 | 1.72 | 2.74 | <.0001 |  |
| Missing/Untreated | 21 | 1.69 | 0.80 | 3.57 | 0.17 |  |
| **Conditioning regimen** |  |  |  |  |  |  |
| Flu/Bu ± TBI | 381 | 1 |  |  |  | 0.0005 |
| Flu/Cy ± TBI | 363 | 1.02 | 0.75 | 1.38 | 0.92 |  |
| Flu/Mel ± TBI | 326 | 1.63 | 1.24 | 2.15 | 0.0005 |  |
| BEAM and similar | 95 | 1.73 | 1.14 | 2.63 | 0.01 |  |
| 2GyTBI ± Flu | 236 | 1.12 | 0.83 | 1.51 | 0.47 |  |
| **GVHD prophylaxis** |  |  |  |  |  |  |
| CNI +MTX (+/- others) | 599 | 1 |  |  |  | 0.04 |
| CNI + MMF (+/- others) | 497 | 1.19 | 0.97 | 1.45 | 0.10 |  |
| CNI + Sirolimus (+/- others) | 275 | 0.74 | 0.53 | 1.03 | 0.08 |  |
| CNI alone | 30 | 1.23 | 0.77 | 1.97 | 0.39 |  |
| **Prior autologous transplant** |  |  |  |  |  |  |
| No | 866 | 1 |  |  |  | 0.01 |
| Yes | 535 | 1.23 | 1.04 | 1.46 | 0.01 |  |

Abbreviations: FL-follicular lymphoma; DLBCL-diffuse large B-cell lymphoma; MCL-mantle cell lymphoma; MZL-marginal zone lymphoma; Flu-fludarabine; Bu-busulfan; Cy-cyclophosphamide; Mel-melphalan; TBI-total body irradiation; CNI-calcineurin inhibitor; MMF-mycophenolate mofetil; MTX-methotrexate; BEAM-Carmustine, etoposide, cytarabine and melphalan; GVHD-graft-versus-host disease; HCT-CI-hematopoietic cell transplantation-Comorbidity index URD-unrelated donor.

*Acute GVHD models used logistic regression.

**Rituximab and disease histology:**

The interaction between main effect (i.e. nonR-RIC vs. R-RIC) and disease histology (DLBCL, FL, MCL, and MZL) was checked for each outcome. It was not significant for grade II-IV acute GVHD (p=0.18), grade III-IV acute GVHD (p=0.64), chronic GVHD (p=0.99), relapse (p=0.96), NRM (p=0.70), PFS (p=0.84) and OS (p=0.53). In addition, multivariate models were constructed for each disease histology (Table S3, S4, and S5). While the sample size in these subgroup models limit the power to detect a statistically significant difference, the relative risks for survival outcomes are consistently in the same direction, as that seen in the multivariate analysis of overall study population (i.e. favoring R-RIC; RR<1.0). The magnitude of relative risk in these subgroup models also suggests a greater degree of benefit of R-RIC for indolent histologies (e.g. Follicular and MCL) and relatively smaller benefit for DLBCL.

**Table S3: Multivariate Analysis Results for Follicular Lymphoma**

|  | **Number** | **Relative Risk** | **95% CI Lower Limit** | **95% CI**  **Upper Limit** | **p-value** |
| --- | --- | --- | --- | --- | --- |
| **Chronic GVHD** |  |  |  |  |  |
| Non-Rituximab RIC | 259 | 1 |  |  | 0.37 |
| Rituximab-containing RIC | 140 | 1.16 | 0.84 | 1.62 |  |
| **Non-Relapse Mortality** |  |  |  |  |  |
| Non-Rituximab RIC | 253 | 1 |  |  | 0.24 |
| Rituximab-containing RIC | 136 | 0.73 | 0.44 | 1.23 |  |
| **Progression/Relapse** |  |  |  |  |  |
| Non-Rituximab RIC | 253 | 1 |  |  | 0.48 |
| Rituximab-containing RIC | 136 | 0.83 | 0.49 | 1.40 |  |
| **PFS** |  |  |  |  |  |
| Non-Rituximab RIC | 253 | 1 |  |  | 0.36 |
| Rituximab-containing RIC | 136 | 0.84 | 1.22 | 0.36 |  |
| **Mortality** |  |  |  |  |  |
| Non-Rituximab RIC | 268 | 1 |  |  | 0.09 |
| Rituximab-containing RIC | 142 | 0.70 | 0.47 | 1.06 |  |

Abbreviations: GVHD=graft-versus-host disease; CI=confidence interval; RIC=reduced intensity conditioning; PFS=progression-free survival; OS=overall survival

**Table S4: Multivariate Analysis Results for Mantle Cell Lymphoma**

|  | **Number** | **Relative Risk** | **95% CI Lower Limit** | **95% CI**  **Upper Limit** | **p-value** |
| --- | --- | --- | --- | --- | --- |
| **Chronic GVHD** |  |  |  |  |  |
| Non-Rituximab RIC | 263 | 1 |  |  | 0.29 |
| Rituximab-containing RIC | 102 | 1.22 | 0.84 | 1.78 |  |
| **Non-Relapse Mortality** |  |  |  |  |  |
| Non-Rituximab RIC | 267 | 1 |  |  | 0.10 |
| Rituximab-containing RIC | 103 | 0.62 | 0.35 | 1.10 |  |
| **Progression/Relapse** |  |  |  |  |  |
| Non-Rituximab RIC | 267 | 1 |  |  | 0.27 |
| Rituximab-containing RIC | 103 | 0.80 | 0.53 | 1.19 |  |
| **PFS** |  |  |  |  |  |
| Non-Rituximab RIC | 267 | 1 |  |  | 0.34 |
| Rituximab-containing RIC | 103 | 0.86 | 0.63 | 1.17 |  |
| **Mortality** |  |  |  |  |  |
| Non-Rituximab RIC | 274 | 1 |  |  | 0.39 |
| Rituximab-containing RIC | 106 | 0.84 | 0.56 | 1.25 |  |

Abbreviations: GVHD=graft-versus-host disease; CI=confidence interval; RIC=reduced intensity conditioning; PFS=progression-free survival; OS=overall survival

**Table S5: Multivariate Analysis Results for Diffuse Large B-cell Lymphoma**

|  | **Number** | **Relative Risk** | **95% CI Lower Limit** | **95% CI**  **Upper Limit** | **p-value** |
| --- | --- | --- | --- | --- | --- |
| **Chronic GVHD** |  |  |  |  |  |
| Non-Rituximab RIC | 433 | 1 |  |  | 0.65 |
| Rituximab-containing RIC | 120 | 1.07 | 0.80 | 1.43 |  |
| **Non-Relapse Mortality** |  |  |  |  |  |
| Non-Rituximab RIC | 443 | 1 |  |  | 0.39 |
| Rituximab-containing RIC | 121 | 1.22 | 0.78 | 1.92 |  |
| **Progression/Relapse** |  |  |  |  |  |
| Non-Rituximab RIC | 443 | 1 |  |  | 0.13 |
| Rituximab-containing RIC | 121 | 0.76 | 0.53 | 1.08 |  |
| **PFS** |  |  |  |  |  |
| Non-Rituximab RIC | 443 | 1 |  |  | 0.48 |
| Rituximab-containing RIC | 121 | 0.90 | 0.68 | 1.20 |  |
| **Mortality** |  |  |  |  |  |
| Non-Rituximab RIC | 452 | 1 |  |  | 0.59 |
| Rituximab-containing RIC | 124 | 0.92 | 0.68 | 1.25 |  |

Abbreviations: GVHD=graft-versus-host disease; CI=confidence interval; RIC=reduced intensity conditioning; PFS=progression-free survival; OS=overall survival

**Table S6: Univariate analysis – Chemorefractory patients**

|  | **Non Rituximab-RIC**  **N=124** | | **Rituximab-containing RIC**  **N=59** | |  |
| --- | --- | --- | --- | --- | --- |
| **Outcomes** | **N Eval** | **Prob (95% CI)** | **N Eval** | **Prob (95% CI)** | **P-value** |
| **NRM** | **116** |  | **56** |  |  |
| 1-year |  | 14 (8-21)% |  | 14 (6-25)% | 0.94 |
| 2-year |  | 19 (12-26)% |  | 16 (8-27)% | 0.67 |
| **Relapse/Progression** | **116** |  | **56** |  |  |
| 1-year |  | 49 (40-58)% |  | 45 (32-58)% | 0.57 |
| 2-year |  | 53 (44-62)% |  | 50 (37-63)% | 0.72 |
| **PFS** | **116** |  | **56** |  |  |
| 1-year |  | 37 (28-46)% |  | 41 (29-54)% | 0.59 |
| 2-year |  | 28 (20-37)% |  | 34 (22-47)% | 0.47 |
| **Mortality (inverse of OS)** | **124** |  | **59** |  |  |
| 1-year |  | 55 (47-64)% |  | 53 (40-65)% | 0.72 |
| 2-year |  | 43 (34-52)% |  | 44 (32-57)% | 0.87 |

**Table S7: Causes of Death**

| **Cause of death** | **Non-Rituximab RIC**  **N (%)** | **Rituximab-containing RIC N (%)** |
| --- | --- | --- |
| Number of deaths | 444 (43) | 139 (37) |
| Graft Rejection | 0 (0) | 1 (<1) |
| Infection | 29 (3) | 13 (3) |
| Idiopathic pneumonia syndrome | 1 (<1) | 2 (<1) |
| Acute Respiratory Distress Syndrome | 2 (<1) | 0 (0) |
| Graft versus Host disease | 36 (4) | 21 (6) |
| Primary disease | 186 (18) | 52 (14) |
| Organ failure | 23 (2) | 7 (2) |
| Second malignancy | 6 (<1) | 2 (<1) |
| Miscellaneous | 143 (14) | 34 (9) |
| Unknown | 18 (2) | 7 (2) |

**Table S8: Studies incorporating rituximab to allo-HCT conditioning regimens in B-cell NHL**

| **Author/ year** | **Study type** | **N** | **Preparative regimens** | **Cumulative Rituximab dose (mg/m^2^)** | **aGVHD (II-IV) Day +100*** | **cGVHD*** | **NRM*** | **Survival outcomes** |
| --- | --- | --- | --- | --- | --- | --- | --- | --- |
| Khouri 2008 | Single arm, prospective, phase 2 | 47 | FLU/CY/R | 3375 | 11% | 60% | 15% | 5-year PFS=83%  5-year OS=85% |
| Pidala 2011 | Retrospective case series | 19 | FLU/BU/R ±ATG | 750 | 58% | 50% | - | 1-year OS=67% |
| Kharfan-Dabaja 2013 | Single arm, phase 2 | 42 | PEN/BU/R^1^ | 1875 | 59% | 69%  (2 yr) | 17% (2 yr) | 2-year PFS=55%  2-year OS=68% |
| Sauter 2014 | Single arm, prospective, phase 2 | 51^2^ | FLU/CY/TBI/R^3^ | 1875 | 18% | 29% (2 yr) | 13% (2 yr) | 2-year EFS=78%  2-year OS=72% |
| Laport 2016 | Multicenter, prospective phase 2 | 62 | FLU/CY/R | 3375 | 27% | 61% (2 yr) | 16% (2 yr) | 2-year PFS=75%  2-year OS=83% |
| Kennedy 2016 | Single center retrospective | 94 | FLU/CY/R^4^ | 3375 | 42% | 24% (2 yr) | 15% (2 yr) | 2-year PFS=58%  2-year OS=73% |

Abbreviations: aGVHD=acute graft versus host disease; ATG=antithymocyte globulin; cGVHD= chronic graft versus host disease; BU=busulfan; CY= cyclophosphamide; FLU= fludarabine; NRM=non-relapse mortality; PEN= pentostatin; R=rituximab; TBI= total body irradiation; PFS= progression-free survival; OS= overall survival

*Depicts cumulative incidence
1. Rituximab was administered only to 33 (79%) patients with CD20^+^ expressing lymphomas
2. Only 49 patients were evaluable

3. Dose of TBI was 200cGy; Rituximab was administered on day -8 and weekly X4 doses beginning D+21
4. Rituximab was administered only to 33 patients (FCR)

**Table S9: Details of GVHD prophylaxis regimens.**

| **Variable** | **No Rituximab** | **Rituximab** |
| --- | --- | --- |
| **CNI + MTX +/- others (except MMF/Siro)** |  |  |
| CNI + MTX | 383 | 206 |
| CNI + MTX + ECP | 2 | 0 |
| CNI + MTX + Corticosteroids | 7 | 1 |
| **CNI + MMF +/- others (except Siro)** |  |  |
| CNI + MMF | 363 | 92 |
| CNI + MMF + MTX | 35 | 2 |
| CNI + MMF + ECP | 2 | 1 |
| CNI + MMF + Corticosteroids | 2 | 0 |
| **CNI+ Sirolimus +/- others** |  |  |
| CNI + Sirolimus | 94 | 42 |
| CNI + Sirolimus + MTX | 97 | 29 |
| CNI + Sirolimus + MMF | 7 | 4 |
| CNI + Sirolimus + ECP | 2 | 0 |

Abbreviations: CNI=calcineurin inhibitor; MTX=methotrexate; ECP=extracorporeal photopheresis; MMF=mycophenolate mofetil; Siro=sirolimus.
